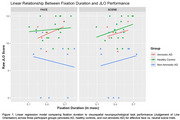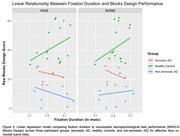# Investigating Eye Movement Metrics and Traditional Visuospatial Task Performance in Heterogeneous Alzheimer's Disease

**DOI:** 10.1002/alz70857_100357

**Published:** 2025-12-24

**Authors:** Hae Young H Yi, Bronte Ficek‐Tani, Corey Horien, Carolyn A Fredericks

**Affiliations:** ^1^ Yale School of Medicine, New Haven, CT, USA; ^2^ University of Washington School of Medicine, Seattle, WA, USA; ^3^ Perelman School of Medicine at the University of Pennsylvania, Philadelphia, PA, USA; ^4^ Department of Neurology, Yale‐New Haven Hospital, New Haven, CT, USA

## Abstract

**Background:**

Eye movement degradation occurs early in Alzheimer's disease (AD). Visual search paradigms demonstrate that AD patients show slower reaction times, greater time to first fixation, and longer fixation durations than controls. Such paradigms may be especially important for evaluating impairment in patients unable to complete traditional pen‐and‐paper neuropsychological assessments. We sought to evaluate the relationship between eye movement metrics and performance on traditional tests of visuospatial function, and investigate whether these relationships differed depending on stimulus category (affective face or neutral scene).

**Method:**

We collected in‐scanner eye‐tracking data (area of interest (AOI) dwell time, fixation duration, and saccade amplitude) while participants freely viewed images of neutral scenes and affective faces, and out‐of‐scanner measures of spatial function and general cognition (WAIS‐III Blocks Design, Judgement of Line Orientation (JLO), Hooper Visual Organization Test (HVOT), and MMSE) in individuals with clinically heterogeneous AD (5 amnestic AD, 10 non‐amnestic AD) and age‐matched healthy controls (*n* = 19). We compared groups using one‐way ANOVA, Kruskal‐Wallis tests, and post‐hoc pairwise t‐tests as appropriate. Linear regression was used to compute the relationship between eye movement metrics and cognitive performance.

**Result:**

As expected, amnestic AD participants underperformed on nearly all neuropsychological metrics compared to HC. Non‐amnestic AD participants performed significantly worse than amnestic AD and HC participants across tests of visuospatial function. We found no group differences in any eye‐tracking metric, regardless of stimulus, or in the overall relationship between eye‐tracking metrics and cognitive testing results. In non‐amnestic AD participants, fixation duration was negatively correlated with JLO and Block Design scores in face trials, but was positively correlated in scene trials.

**Conclusion:**

Non‐amnestic AD participants showed different relationships between fixation duration and visuospatial function scores for face vs. scene trials, suggesting that more frequent sampling of regions of an affective face may improve visuospatial task performance in non‐amnestic AD participants. While preliminary, these findings suggest eye movement metrics may add meaningful information to our interpretation of neuropsychological test performance, and highlight the importance of considering eye movement performance in a clinical context.